# Asymmetric visual input and route recapitulation in homing pigeons

**DOI:** 10.1098/rspb.2015.1957

**Published:** 2015-10-07

**Authors:** Antone Martinho, Dora Biro, Tim Guilford, Anna Gagliardo, Alex Kacelnik

**Affiliations:** 1 Department of Zoology, University of Oxford, South Parks Road, Oxford OX1 3PS, UK; 2 Department of Biology, University of Pisa, Via Volta 6, Pisa 56126, Italy

**Keywords:** *Columba livia*, homing, tecto-fugal pathway, monocular route learning, route recapitulation, visual asymmetry and learning

## Abstract

Pigeons (*Columba livia*) display reliable homing behaviour, but their homing routes from familiar release points are individually idiosyncratic and tightly recapitulated, suggesting that learning plays a role in route establishment. In light of the fact that routes are learned, and that both ascending and descending visual pathways share visual inputs from each eye asymmetrically to the brain hemispheres, we investigated how information from each eye contributes to route establishment, and how information input is shared between left and right neural systems. Using on-board global positioning system loggers, we tested 12 pigeons' route fidelity when switching from learning a route with one eye to homing with the other, and back, in an A-B-A design. Two groups of birds, trained first with the left or first with the right eye, formed new idiosyncratic routes after switching eyes, but those that flew first with the left eye formed these routes nearer to their original routes. This confirms that vision plays a major role in homing from familiar sites and exposes a behavioural consequence of neuroanatomical asymmetry whose ontogeny is better understood than its functional significance.

## 1. Introduction

Birds and mammals moving in familiar landscapes acquire, process, store and use visual information, but while in the mammalian brain bilateral projections from the optic chiasma and interhemispheric connectivity through the corpus callosum ensure that visual information is readily available bilaterally, in birds visual pathways are almost completely decussated at the optic chiasm, and there is no corpus callosum. These differences make integration of visual input from the two eyes very different between these two vertebrate classes, and lead to differences in learning and memory [1]. Pigeons using visual landmarks to fly homewards from familiar locations [2] provide an opportunity to study how visual information acquired by each eye is stored and made available to contralateral visual control of navigation.

How pigeons find their way home has been subject of extensive research and lively debate for many years [3,4]. Field experiments have shown that pigeons compute their homeward bearing based on a ‘map’ (information on the bird's position relative to the loft, probably derived from olfactory cues when in unfamiliar territory) [2,3,5] and use a time-sensitive sun compass [6,7] or a magnetic compass [8] in assuming a homeward bearing and navigating to their loft. As they acquire experience with an area, pigeons progressively incorporate visual cues in the form of landmarks to engage in pilotage—or navigation by a series of visual landmarks—with reliance on compass information decreasing with experience [9–12]. On-board global positioning system (GPS) loggers have greatly enriched the study of pigeon homing, revealing that pigeons released repeatedly from the same site form individually idiosyncratic routes that are tightly recapitulated on subsequent flights [10,11,13]. Furthermore, their routes often incorporate long linear landmarks such as roads, railway lines and rivers [9].

The pigeon visual system is lateralized at both neuroanatomical and functional level, as shown in birds tested in laboratory cognitive tasks [14]. Functional asymmetries have also been found for spatial tasks involving memorization and processing of cues in both laboratory settings and natural behaviours, such as homing [15]. In particular, the functional contribution of the left and right side of the brain in homing behaviour has been the subject of experiments focusing on the hippocampal formation and olfactory and visual systems [15,16]. Although both the left and right parts of the hippocampal formation seem to be involved in familiar landmark-based navigation [17], an intact left hippocampus is needed for olfactory map learning in young pigeons raised confined [18], probably due to the critical role of the left hippocampus in sun compass-mediated spatial learning [19] through which the association of wind-born odours with wind direction is memorized. In the olfactory system, an asymmetric involvement in favour of the left piriform cortex and of the right nostril has been highlighted [15].

Within the visual system, pigeons' optic nerves completely decussate at the optic chiasm, projecting exclusively to the tectum contralateral to the input eye [20], meaning that direct visual inputs to a hemisphere can be eliminated by capping an eye. The ascending tecto-fugal and thalamo-fugal visual pathways do allow for indirect visual input to the contralateral hemisphere [21], but while each tectum projects to the ipsilateral diencephalic nucleus rotundus roughly equally, more projections from the right tectum are shared contralaterally to the left nucleus rotundus and entopallium than from left to right [1,14,22]. This asymmetry is developmentally related to the orientation of the pigeon embryo, whereby the right eye is exposed to more light during incubation [14,23]. The importance of this light exposure to interhemispheric integration has been shown in visual transitive inference tasks performed by monocularly occluded pigeons [20]. Memory formation and storage may be lateralized, with predominance of the left hemisphere [24], and further asymmetries of projections have been found in both the ascending and descending visual systems [25]. This fits into the wider model of the avian visual system as being lateralized differently for specific tasks and roles [26]. Nonetheless, this model is largely derived from chickens, and it is likely that detailed patterns of lateralization differ between pigeons, chickens and other birds.

**Figure 1.**
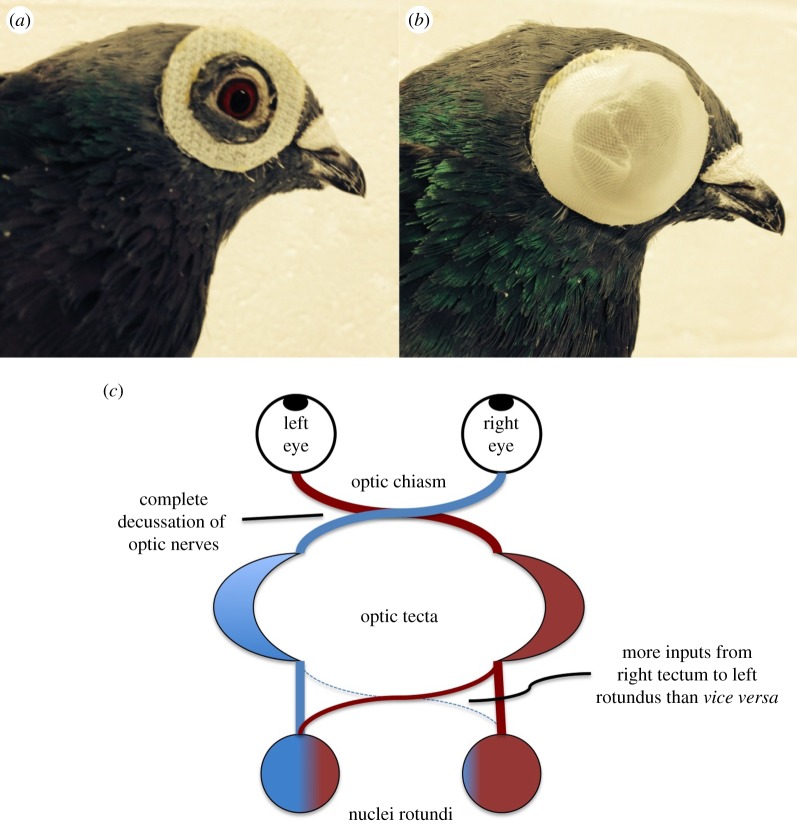
Pigeon with eye ring and eye cap, and generalized structure of pigeon tecto-fugal system. (*a*) Each pigeon was fitted with two eye rings as shown. The ‘hook’ side of Velcro was used for the ring attached to the pigeons' feathers as its thinner profile ensured forward vision would not be compromised. (*b*) The eye caps were constructed of a double layer of flexible etched plastic. The seam in the eye cap was consistently oriented posteriorly to ensure a homogeneous image in the forward direction. (*c*) Schematic of the pigeon tecto-fugal visual system, detailing the asymmetry of inputs. Adapted from [23].

Here, we investigate whether the asymmetries of the visual memory system are reflected in pigeons' route fidelity when switching from monocular homing with an ‘experienced’ to a ‘naive’ eye. We trained 12 pigeons to fly home under reversible, non-invasive monocular occlusion (figure 1) for 18 flights before subsequently flying another 18 flights from the same release point with the opposite eye blocked. This was followed by five flights with the original eye blocked, and finally by five binocular flights, completing a total of 46 flights per bird, in four phases. Our results reflect a poverty of interhemispheric exchange of visual homing information and a hemispheric imbalance of information availability in the pigeon brain.

## 2. Results

The 12 experimental birds flew in two groups defined by the sequence of which eye was available (i.e. the eye which was not occluded) in each phase of experimentation: ‘left–right–left–binocular’ (hereafter LRLB) and ‘right–left–right–binocular’ (hereafter RLRB). A control group of four birds flew 18 non-occluded flights. Positional fixes recorded at 5 Hz by on-board GPS loggers allowed us to reconstruct the pigeons' flights (see ‘Experimental procedures’) and to compare paths flown. The set of the first 18 flights for each group is referred to as ‘phase 1’ and the second 18 flights, flown with the other eye, as ‘phase 2’. The subsequent five flights, flown with the original eye, comprise ‘phase 3’. For clarity, except where otherwise indicated, the descriptors ‘left eye’ and ‘right eye’ refer to the eye that is open (not occluded) for the flight being described.

**Figure 2.**
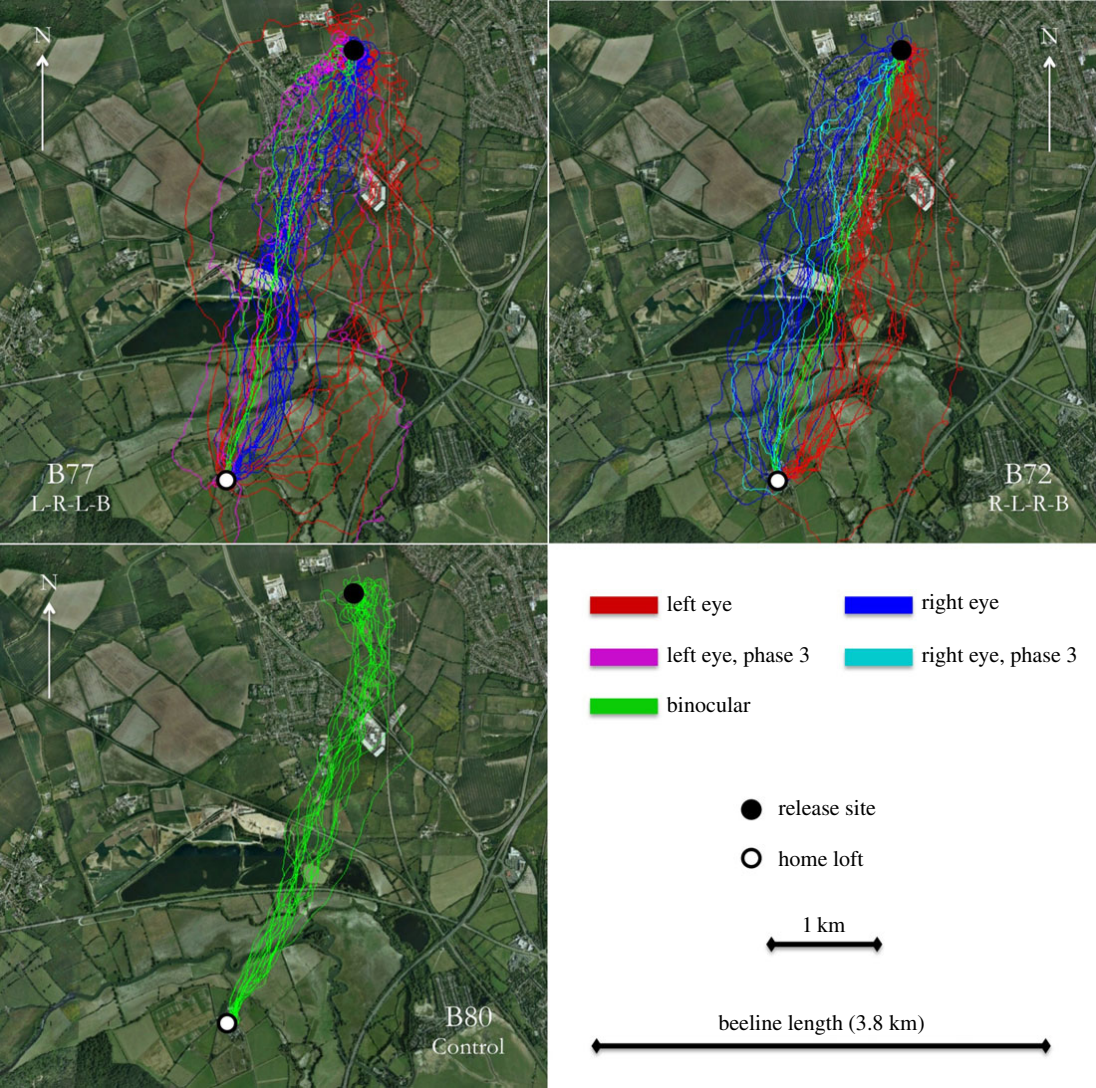
Examples of flight series for two experimental and one control pigeon. Each experimental pigeon flew 46 flights in either a ‘18 left, 18 right, 5 left, 5 binocular’ or ‘18 right, 18 left, 5 right, 5 binocular’ pattern (referring to the eye open, not the eye occluded). Bird IDs and treatment received are indicated in the bottom right corner of each panel. Left-eyed flights are shown in red (magenta for phase 3, bird B77 only), whereas right-eyed flights are blue (cyan for phase 3, bird B72 only). Binocular flights are shown in green. The control pigeons flew 18 flights each, all of which were binocular.

**Figure 3.**
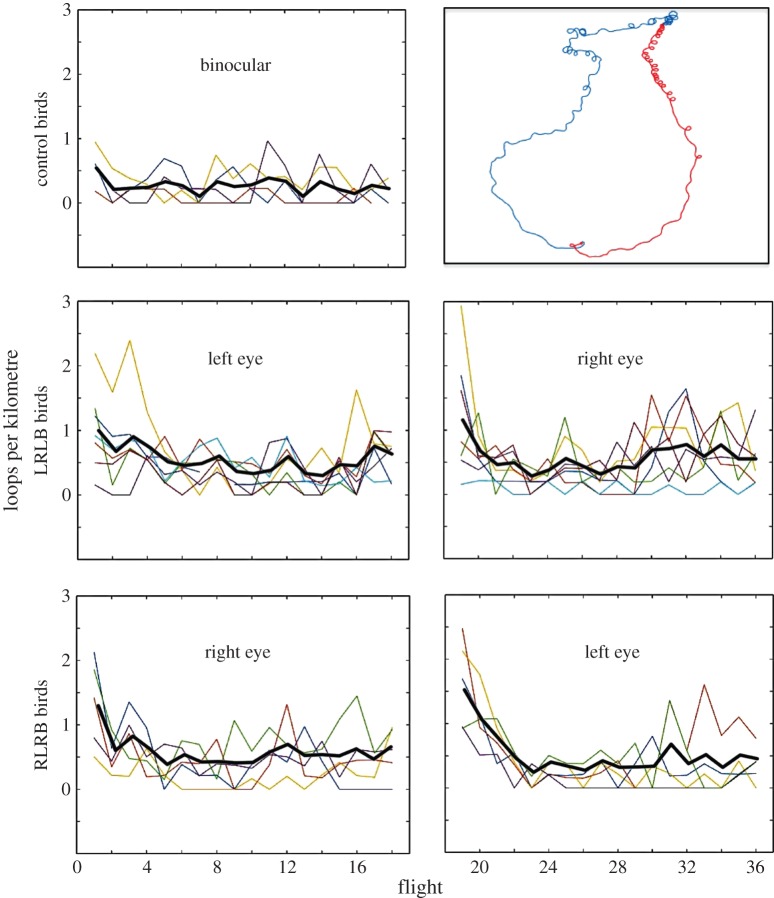
Looping rate analysis. Monocularly occluded birds perform tight loops (approx. 25–50 m in diameter) around the open eye during early flights as seen in the panel at top right (the blue track is a right-eyed bird, the red, left). The binocular controls and both phases of experimental flights show significant decrease in looping rate throughout the 18 flights. The heavy black lines show the mean value across pigeons in the group for each flight. High early looping rates may reflect information gathering by visually hemicompromised pigeons.

Visual inspection of the trajectories revealed consistent patterns, as displayed by exemplars in figure 2. For all experimental birds, the first several flights show properties distinct from subsequent monocular flights and from all control flights. In these early monocular flights, the pigeons' routes deviate from the beeline between the release site and the loft in the direction of the available eye. As the pigeons become more experienced with a given eye, the routes migrate towards the beeline. Furthermore, during early flights, the pigeons often perform many tight loops around the open eye—clockwise when the right eye is open and counterclockwise when the left eye is open—possibly attempting to gather information from a 360° field of view. This is a behaviour not previously described, as far we are aware. At no point in the study was a bird seen to loop around an occluded eye. Control birds did perform loops, but as both eyes were open, flights included loops in both directions. We quantified each bird's looping rate as loops per kilometre of route flown, excluding loops made around the release site and home loft (figure 3). Among experimental birds, looping rate begins high and subsides through subsequent flights, before returning to a high rate when the occluded eye is switched, and subsiding in a similar pattern. We compared the looping rate across flights within each of the controls, and the first two phases of each of the LRLB and RLRB birds, using a two-way repeated measures ANOVA. The 18 flight phases of the experimental birds were considered separately, such that the ANOVA included five groups of 18 repeated measures (flights). The rate of looping differs significantly across flights (*F*_17,340_ = 7.162, *p* < 0.001), indicating that reduced looping rates correlate with increased experience homing over a given route. There was no significant interaction between looping rate and group, indicating that this effect is similar across groups. Both experimental groups showed an increase in looping rate following the first eye switch followed by a decline with experience. The cause for this looping may simply be that it takes time to adjust to the new circumstances, or, more interestingly, that using an eye that is naive for the release site, in the absence of interocular transfer, requires learning of new visual landmarks, and that looping in the direction of the naive eye reflects this search for visuo-spatial input. One possible mechanism in either case may be that under binocular conditions, the view of each eye ‘pulls' the animal to the corresponding side, and being newly deprived of one eye's counterbalance results in a loop, until this is corrected by experience.

**Figure 4.**
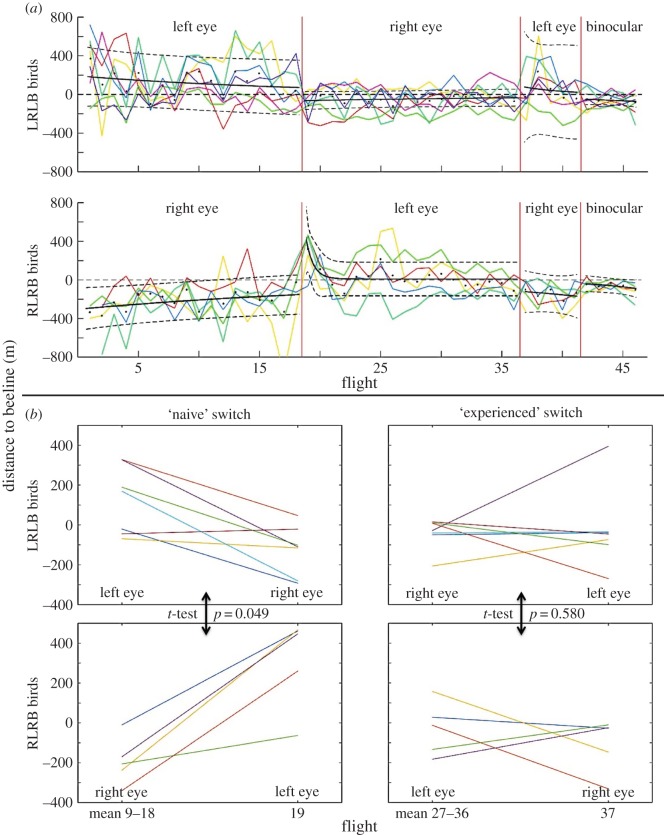
Beeline analysis. The beeline analysis calculates the distance to the beeline for each point along a flight and gives the mean of these distances as the score for that flight. (*a*) Each bird's score is shown for each flight. The black lines show a fitted exponential curve with 95% confidence intervals (fitted parameters are given in the electronic supplementary material). The ‘left eye’ and ‘right eye’ labels refer to the open eye. Points to the right of the beeline, with reference to the direction of motion, are scored as negative values, while those to the left are positive. (*b*) For each of the two eye-switches from phase 1 to phase 2 and phase 2 to phase 3, the mean of the final 10 flights before the switch is compared with the first flight after the switch.

To test whether information input to the contralateral hemisphere was more effective in one direction than the other, we assessed how the birds' trajectories changed across phase changes. To do this, we used a distance-to-beeline analysis (figure 4). Each flight was sampled at points representing GPS positional fixes collected at 5 Hz. This analysis computes the mean perpendicular distance in metres between the beeline and each point in a given flight, assigning positive values to scores left of the beeline and negative values to those to the right, with respect to the direction of flight. Figure 4*a* shows the beeline analysis for each flight. To compare the degree to which routes changed across phases, we computed the difference in distance to beeline between each bird's first flight after an eye switch and the mean of the 10 flights before the switch (figure 4*b*), for both the initial switch from phase 1 to phase 2 (i.e. switching to a ‘naive’ eye) and the subsequent switch from phase 2 to phase 3 (i.e. switching back to the eye that became ‘experienced’ in phase 1). We calculated the magnitude of the change from pre-switch mean to the first post-switch flight for each bird and compared the groups via two-sample *t*-tests, as shown in figure 4*b*. The average (±s.e.) signed magnitude of the changes in distance to the beeline after the first (naive) switch was −250.2 ± 67.0 m for the LRLB group and 506.9 ± 98.5 m for the RLRB group. In addition to the obvious sign difference (figure 4*b*), the absolute value of the difference between the first and second phase for the RLRB group was significantly greater than for the LRLB group (*p* = 0.049, two-sample *t*-test), corresponding with the move across the beeline seen in the RLRB graph, indicating that the asymmetric effect of the treatment was true at individual level and not an artefact of averaging. This implies that the LRLB group's route was less affected by the eye switch than was the case for the RLRB group. It is possible that information concerning a general spatial affinity for the landmarks acquired with the left eye/right hemisphere system (LRLB group) reached the left hemisphere via an indirect input to a greater degree than in the opposite direction (RLRB group). There was no significant difference between groups in the absolute value of change between the second and third phases (*p* = 0.580).

**Figure 5.**
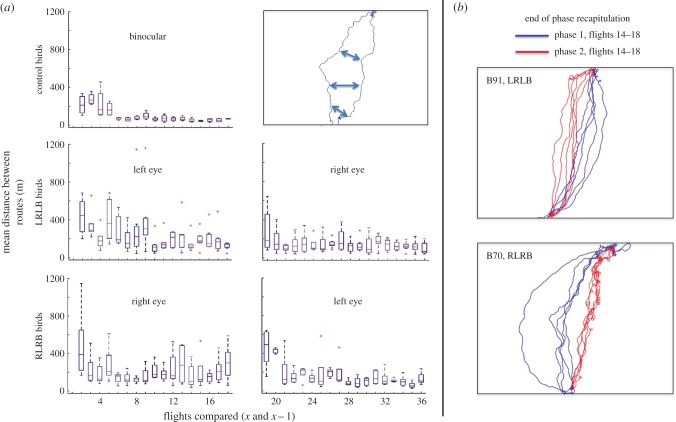
Nearest neighbour analysis of route development. (*a*) The nearest neighbour analysis calculates the degree to which a flight has changed with respect to the previous flight, as seen in the panel at top right (see ‘Experimental procedures’ for details). The blue arrows represent the shortest distances between the two flights, computed at each point in the flight (here, three are shown for clarity). The pairwise comparisons of flights in each group (controls, LRLB and RLRB) are shown as boxplots about the median. The ‘left eye’ and ‘right eye’ labels refer to the open eye. (Note that since there is no flight previous to the first, the first frame is left empty.) Recapitulation occurs when nearest neighbour values remain low, while adjacent high values indicate variability of route. (*b*) Examples showing that both LRLB and RLRB birds recapitulated over a different route in each phase.

While the LRLB birds did fly nearer to their phase 1 routes in phase 2 than was the case for RLRB birds, the beeline analysis does not determine the fidelity of route recapitulation (similar mean distance to the beeline can result from different trajectories). For this reason, route fidelity was examined using a ‘nearest neighbour analysis' (figure 5*a*), in which each flight within a bird's series was compared to the immediately preceding flight. The details of this analysis are presented in the ‘Experimental procedures’ section below. The nearest neighbour analysis shows how the route differs between consecutive pairs of flights [27], providing a measure of variability or fidelity of a bird's recapitulation of its route. Figure 5*a* shows the nearest neighbour results for the controls and the first phases of the two experimental groups. The controls show a rapid progression from early variability to asymptotic fidelity. These results were compared to the first phase of both experimental groups via two-way repeated-measures ANOVAs. All three groups showed a significant main effect of decrease in nearest neighbour distance with experience across the 17 pairwise comparisons (*F*_16,176_ = 1.827, *p* = 0.031), but no significant difference between the groups (*F*_32,176_ = 0.876, *p* = 0.662). The phase 2 results of the experimental groups were compared in the same way, with both groups again showing a significant decrease across the 18 pairwise comparisons (including the cross-phase comparison of the last flight in phase 1 to the first in phase 2; *F*_17,170_ = 7.098, *p* < 0.001), and a significant difference in nearest neighbour values between the two groups (*F*_17,170_ = 2.167, *p* = 0.007). This is consistent with the results of the beeline analysis, which showed that the RLRB group displayed a larger shift in spatial location of route across the eye switch. Both groups were affected by the switch from the first eye to the second—the high nearest neighbour values imply that the first flight of phase 2 did not recapitulate the last flight of phase 1 in either group. This is consistent with the findings of the beeline analysis, which showed that both groups' routes changed, albeit to different degrees, across the initial eye switch. This implies that while both groups achieved consistent recapitulation by the end of each of phases 1 and 2, both groups also modified their routes after the switch, as nearest neighbour distances increased at the beginning of phase 2.

The high nearest neighbour values at the beginning of phase 2 do not exclude the possibility that the birds produced high variability as a result of the eye switch, but then settled back to the same recapitulated route as before the switch—especially the LRLB birds, which were shown in the beeline analysis to achieve similar scores across phases. However, as we noted earlier, similarity of beeline score does not imply route recapitulation, and a visual inspection of the final five flights for each bird in each of phases 1 and 2 reveals that this was not the case. Figure 5*b* shows representative examples of these flights for each group (equivalent figures for all birds are supplied in electronic supplementary material, figure S1). As can be seen, while within phase flights are near to each other, routes differ across phases. Some birds, especially in the LRLB group, did fly very near their previous route, but the shape of their route differed between the two phases. While the LRLB birds did show slightly higher route fidelity after the eye switch than the RLRB birds, the complete information necessary for precise recapitulation, perhaps including sequence, bearings or other onward guidance connecting one landmark to another, was not sufficiently available to either group to allow strict recapitulation.

## 3. Discussion

Our results indicate that in pigeons input of visual homing information from each eye is not closely integrated with input from the other eye, that the level of integration is asymmetric and that this asymmetry has functional consequences. Pigeons' routes learned solely with one eye are not recapitulated when flying with the other, naive eye, and this failure to recapitulate occurs regardless of which eye learns first, though birds that home first monocularly with the left eye fly significantly nearer to their left-eyed route when subsequently flying right-eyed than vice versa. This suggests that the various commissures in the pigeon visual pathway allow insufficient contralateral input of route information for strict recapitulation using the naive eye. However, route recapitulation by visual landmarks requires both visual and non-visual memory, as a pigeon must both recognize each landmark visually and remember which direction to take and which landmark comes next in sequence at each point to follow its established route tightly. It is thus possible that though this strict sequence was lost in both groups, the LRLB group's more general visual recognition and attraction to the previously learned landmarks may have endured across phases and may reflect the asymmetry of the pigeon's visual pathway, especially in the ascending tecto-fugal pathway.

Concordant with the asymmetry present in the commissures of the tecto-fugal visual pathway, birds flying with the right eye after learning initially with the left eye, as seen in the LRLB group, fly significantly nearer to their original (left eye), first-learned route than birds flying in the opposite order. Thus, although neither group using the second eye followed the precise set and sequence of visual landmarks learned with the first eye, the asymmetry of the pigeon visual system [1,14,23] means that information acquired with the left eye is more readily available to control visually guided behaviour by the other eye than the opposite. Even after repeated experience, the phase 2 routes of the RLRB group settle farther from their initial (left-eyed acquired) routes than those of the LRLB birds, possibly because the weaker projections from the left optic tectum to the right nucleus rotundus are much reduced relative to the opposite side [1,14,23], though other neural asymmetries may contribute to this difference [25]. Thus, though neither eye can access the exact sequence of landmarks determining the route established with the other eye, birds flying right-eyed after left fly more closely to the routes learned with the left eye. The fact that no significant change in route was found in either group upon returning to the first eye in the third phase is consistent with previous observations that pigeons retain learned recapitulated routes over long periods of time. In both groups, by the beginning of the third phase, 18 flights had been flown by each eye, thus allowing whichever eye was used in the third phase to access those visual memories and home accordingly.

Our beeline analysis results further indicate that the difference in performance cannot be solely attributed to lateralization—the superiority of one eye or hemisphere over the other for homing performance. If this were the case, we would expect to find similar performance for a given eye across both groups in all phases. Instead, we see that homing performance for a given eye varies depending on the sequence of training and previous experience. This suggests that our results derive from the asymmetry of visual inputs to the two hemispheres, rather than simple superiority of one hemisphere or eye.

These results confirm the suspected effects of neuroanatomical division and asymmetry of visual processing, and contribute to our understanding of visual lateralization in homing for pigeons with some homing experience. A previous study on monocular homing inferred the importance of the eyes for homing lateralization from different homing speed and vanishing bearings with each eye, but argued that since both naive and experienced birds showed vanishing bearing deflection and lateralization of performance, this was not an effect of visual memory but potentially of lateral specialization for magneto-reception or optic flow [28]. However, naive and experienced pigeons navigate by different strategies; namely, naive pigeons rely mostly on site-specific compass orientation, whereas experienced pigeons transition to pilotage-based orientation within the familiar area [29]. By using GPS technology that allows investigation of the fidelity of homing to previously recapitulated routes, we have shown that the better performance of the LRLB pigeons in phase 2 (when flying with the previously naive right eye) is likely to be a result of the left hemisphere's more complete visual memory of homing routes. The phase 2 results indicate that pigeons experienced with both eyes separately show right eye/left hemisphere superiority in homing, a pattern previously noted in pigeons flying monocularly after simultaneous binocular experience [30], and in pigeons navigating in an indoor arena strewn with visual landmarks [31].

Finally, our results reaffirm the importance of vision itself in the process of pigeon homing. At one time the subject of debate, the degree of relevance of visual information in allowing pigeons to reliably home remains in question. While the formation of, and loyalty to, individually idiosyncratic homing routes have been taken as key support for the importance of visual information and memory in familiar area homing [9], these inferences were indirect. The current study is the first to assess route recapitulation while manipulating visual information input directly during homing, and demonstrates that monocular homing performance follows well the pattern established in other purely visual tasks. Pigeons undertaking a monocular binary discrimination task after binocular training show better performance with the right eye than with the left, again probably a consequence of the right eye's access to a more complete visual memory [32]. Our results demonstrate a similar pattern for familiar area homing, and thus situate this homing within the pigeon's visually controlled behaviours.

## 4. Experimental procedures

### (a) Subjects and materials

Sixteen homing pigeons 2 years of age and of both sexes were used. Pigeons were housed at the Oxford University Field Station, Wytham, UK, where they had also been bred. Pigeons were kept at free-feeding weight with unlimited access to water, grit and mixed grain food, and allowed to fly freely from the loft daily.

Twelve experimental birds were prepared for attachment of GPS logging devices by trimming non-flight feathers on their backs and applying a 4 cm strip of Velcro with fast-drying glue. All GPS devices were initially attached via the Velcro method, but in cases in which the strip became loose during the course of the experiment, the attachment method was switched to the use of an elasticated harness (for further details, see ‘Irregularities’ below). Rings of Velcro to facilitate eye cap attachment were glued around each eye as in figure 1. QStarz BT-Q1300ST 5 Hz personal GPS loggers were modified for attachment to pigeons by removing the outer plastic covering. Eye caps (figure 1) consisted of a 2 cm diameter disc composed of a double layer of etched plastic to allow for light penetration but prevent shape identification. The remaining four birds formed a control group and were prepared in the same fashion for GPS attachment, but were not fitted with eye rings.

All pigeons received basic training of at least three flock releases and three solo releases from locations roughly aligned with the four cardinal directions, approximately 2 km from the loft, alternated to prevent directional bias. All training flights were flown binocularly.

### (b) Releases

Releases investigated homing in birds completely naive to the release site. All birds' experimental releases were solo flights and birds were released 7 min apart to prevent *ad hoc* flock formation. The pigeons had no previous homing experience beyond basic training. A single release point was used, but all analyses compared changes in route within subject and between flights, rather than location of route with reference to the landscape. Any bias towards landmarks from this release site is therefore unlikely to have affected our results. Furthermore, the use of a single release site minimized loss of birds.

#### (i) Experimental groups

The 12 experimental birds were divided into two groups, one of seven (LRLB group) and one of five (RLRB group). The groups initially contained eight birds each, but one bird from the LRLB group and three birds from the RLRB group failed to home during the first flight (see ‘Losses and Irregularities’ below).

The pigeons were released from a novel location 3.8 km from the loft. The releases comprised 50 flights in four phases over the course of three months.

Phase 1 (flights 1–20) consisted of first-eye training and testing. The LRLB group flew with their left eye (right eye covered) and the RLRB group with the right eye (left eye covered) during all flights in phase 1. The first 18 of the 20 phase 1 flights completed by each bird were included in analysis. The final two flights were reserves, included to accommodate occasions when a bird missed a flight for one of a number of reasons or a GPS logger failed (see ‘Irregularities’ below). In cases in which a bird did not miss two flights within the set of 20, any flights beyond the 18th (one to two flights) were excluded from consideration.

Phase 2 (flights 21–40) consisted of second-eye training and testing, and followed the same pattern as phase 1. In phase 2, the LRLB group flew with their right eye (left covered) and the RLRB group with their left eye (right covered). As in phase 1, the pigeons flew 20 flights, of which 18 were included in analysis, and two were reserves.

Phase 3 (flights 41–45) consisted of re-testing the first eye. The LRLB group flew with their left eye and the RLRB group with their right eye. All pigeons flew five flights in phase 3.

Phase 4 (flights 46–50) consisted of binocular testing. All 12 pigeons flew five flights with both eyes uncovered.

#### (ii) Control group

The four control birds were released binocularly 18 times from the same site as the experimental birds, following the same protocols. The first 13 flights comprised training and the last five flights (flights 14–18) were used to determine ‘normal high-familiarity homing’ for comparison with experimental birds. The control birds did not experience any irregularities.

### (c) Data processing

GPS traces of flight paths were converted into comma-separated-values format by proprietary software included with the GPS loggers. All subsequent processing and analysis of flight paths was performed in MATLAB. All flights were trimmed for speed (retaining all points for which speed was greater than 0.5 m s^−1^ for 30 s before or after) to exclude waiting time before release and after re-entering the loft. Flights were then trimmed to exclude points within 200 m of the start and endpoints to exclude time when all flights are constrained to be very close together. To assist comparisons with other experiments, a table of commonly used pigeon homing metrics before the advent of GPS location may be found in the electronic supplementary material.

#### (i) Nearest neighbour analysis

The nearest neighbour analysis holds one of the two flights compared in reference, and for each point along the reference measures the distance to the closest point on the compared flight in any direction (figure 5*a*, inset). For each pairwise comparison, both flights are held in reference and compared to the other in turn, and the mean of these two series of distances provides the mean nearest neighbour distance between the two flights.

### (d) Losses and irregularities

#### (i) Losses

The first flight of the experimental releases was performed with a slightly modified version of the eye cap that incorporated a layer of tracing paper to partially occlude light. Four of 16 birds failed to home during this first flight. As a result of this high failure rate, the eye caps were restructured to exclude the tracing paper and include a second layer of etched plastic, preserving the shape disruption but allowing for still more light penetration. Following this modification, the remaining 12 birds homed successfully. Among the 12 birds that successfully homed during the first flight, performance was reduced, with long routes very far from the beeline.

#### (ii) Irregularities

On some occasions, a bird did not produce data during a given flight. When this occurred, a reserve flight was used. All such irregularities occurred during phases 1 and 2, and in no case did a bird require more than two reserve flights. Causes of irregularities included sudden weather changes, GPS device failure and birds not yet returned from free flight around the loft. Additionally, nine birds' Velcro strips fell off during the course of the experiment. As this was a result of feather moulting beneath the strip, the strip could not be reattached until the following season, and so was replaced by a ‘backpack’ consisting of a fabric pouch to hold the GPS logger, attached to the pigeon by elasticated straps that go around the wings and across the keel. In seven of these cases, the bird in question was mounted with the backpack and excluded from flights for the remainder of the day to allow it to become accustomed to the backpack. Each of these birds wore the backpack for the remaining duration of the experiment and during all remaining flights.

## Supplementary Material

Electronic Supplementary Material

## Data accessibility

GPS tracks of all flights in the experiment are available on Dryad: http://dx.doi.org/10.5061/dryad.2c47d.
