# Supplementary material for: Asymmetric visual input and route recapitulation in homing pigeons
Source: Proc Biol Sci. 2015 Oct 7;282(1816):20151957. doi: 10.1098/rspb.2015.1957 (PMC4614786; doi:10.1098/rspb.2015.1957)
Supplement: Electronic Supplementary Material [file rspb20151957supp1.pdf]

Electronic Supplementary Material

Curve fitting details for Figure 4

All curves were fitted using the equation $y = ae^{bx}$.

Coefficients and goodness of fit:

LRLB Flights 1–18

Coefficients (with 95% confidence bounds):
a = 184.8 (25.49, 344.2)
b = -0.05244 (-0.1559, 0.05105)

Goodness of fit:
SSE: 2.271e+05
R-square: 0.06127
Adjusted R-square: 0.002601
RMSE: 119.1

LRLB Flights 19–36

Coefficients (with 95% confidence bounds):
a = -72.33 (-123.6, -21.11)
b = -0.03577 (-0.1135, 0.04197)

Goodness of fit:
SSE: 2.728e+04
R-square: 0.05989
Adjusted R-square: 0.001134
RMSE: 41.29

LRLB Flights 37–41

Coefficients (with 95% confidence bounds):
a = 98.05 (-652.5, 848.6)
b = -0.307 (-3.716, 3.102)

Goodness of fit:
SSE: 4.817e+04
R-square: 0.0742
Adjusted R-square: -0.2344
RMSE: 126.7

LRLB Flights 42–46

Coefficients (with 95% confidence bounds):

a = -49.34 (-89.03, -9.653)
b = 0.08863 (-0.1333, 0.3105)

Goodness of fit:
SSE: 598.7
R-square: 0.3341
Adjusted R-square: 0.1122
RMSE: 14.13

RLRB Flights 1–18

Coefficients (with 95% confidence bounds):
a = -302.6 (-412.4, -192.9)
b = -0.03731 (-0.07745, 0.002829)

Goodness of fit:
SSE: 1.235e+05
R-square: 0.189
Adjusted R-square: 0.1383
RMSE: 87.85

RLRB Flights 19–36

Coefficients (with 95% confidence bounds):
a = 1661 (-3127, 6448)
b = -1.649 (-4.376, 1.077)

Goodness of fit:
SSE: 1.114e+05
R-square: 0.4802
Adjusted R-square: 0.4477
RMSE: 83.46

RLRB Flights 37–41

Coefficients (with 95% confidence bounds):
a = -120.9 (-279.7, 38.02)
b = 0.07576 (-0.2912, 0.4427)

Goodness of fit:
SSE: 9118
R-square: 0.1366
Adjusted R-square: -0.1512
RMSE: 55.13

RLRB Flights 42–46

Coefficients (with 95% confidence bounds):
a = -41.37 (-92.54, 9.797)
b = 0.1723 (-0.1461, 0.4907)

Goodness of fit:
SSE: 1399
R-square: 0.4783
Adjusted R-square: 0.3044
RMSE: 21.59

Classical Pigeon Metrics

Efficiency scores

Route efficiency is calculated as the beeline distance from release point to home loft divided by the total distance travelled. Mean efficiency is given for the selection of flights indicated.

	Phase 1, Flights 1-5	Phase 1, Flights 14-18	All Phase 1 Flights	Phase 2, Flights 1-5	Phase 2, Flights 14-18	All Phase 2 Flights	All Phase 3 Flights	All Phase 4 Flights
LRLB Birds	0.582	0.711	0.669	0.711	0.699	0.709	0.670	0.798
RLRB Birds	0.616	0.705	0.683	0.642	0.784	0.729	0.721	0.792
Controls	0.695	0.759	0.759					

Homing time scores

Homing time is the time, in seconds, from release to landing at loft.

	Phase 1, Flights 1-5	Phase 1, Flights 14-18	All Phase 1 Flights	Phase 2, Flights 1-5	Phase 2, Flights 14-18	All Phase 2 Flights	All Phase 3 Flights	All Phase 4 Flights
LRLB Birds	641.2	307.0	473.2	328.9	352.1	361.7	475.6	439.9
RLRB Birds	510.4	324.6	440.0	373.3	306.1	339.7	379.3	266.7
Controls	251.1	548.3	329.8					

Virtual vanishing bearing scores

Virtual vanishing bearing is the direction of flight, relative to the release point, when the bird first exceeds a distance of 2.5km from the release point. It is expressed in degrees, with 0 or 360 indicating North, 90 East, 180 South, and 270 West

	Phase 1, Flights 1-5	Phase 1, Flights 14-18	All Phase 1 Flights	Phase 2, Flights 1-5	Phase 2, Flights 14-18	All Phase 2 Flights	All Phase 3 Flights	All Phase 4 Flights
LRLB Birds	185.8	192.1	190.3	198.4	196.3	197.6	193.7	196.4
RLRB Birds	225.4	203.3	209.4	189.6	196.6	192.6	201.1	195.1
Controls	196.2	193.7	194.7					

Virtual vanishing time scores

Virtual vanishing time is the time in seconds between release and when the bird reaches the point at which virtual vanishing bearing (above) is taken.

	Phase 1, Flights 1-5	Phase 1, Flights 14-18	All Phase 1 Flights	Phase 2, Flights 1-5	Phase 2, Flights 14-18	All Phase 2 Flights	All Phase 3 Flights	All Phase 4 Flights
LRLB Birds	281.8	189.1	223.5	218.4	246.7	227.6	268.5	185.9
RLRB Birds	310.9	202.0	245.3	253.9	208.1	223.9	317.7	210.0
Controls	199.8	195.6	185.8					

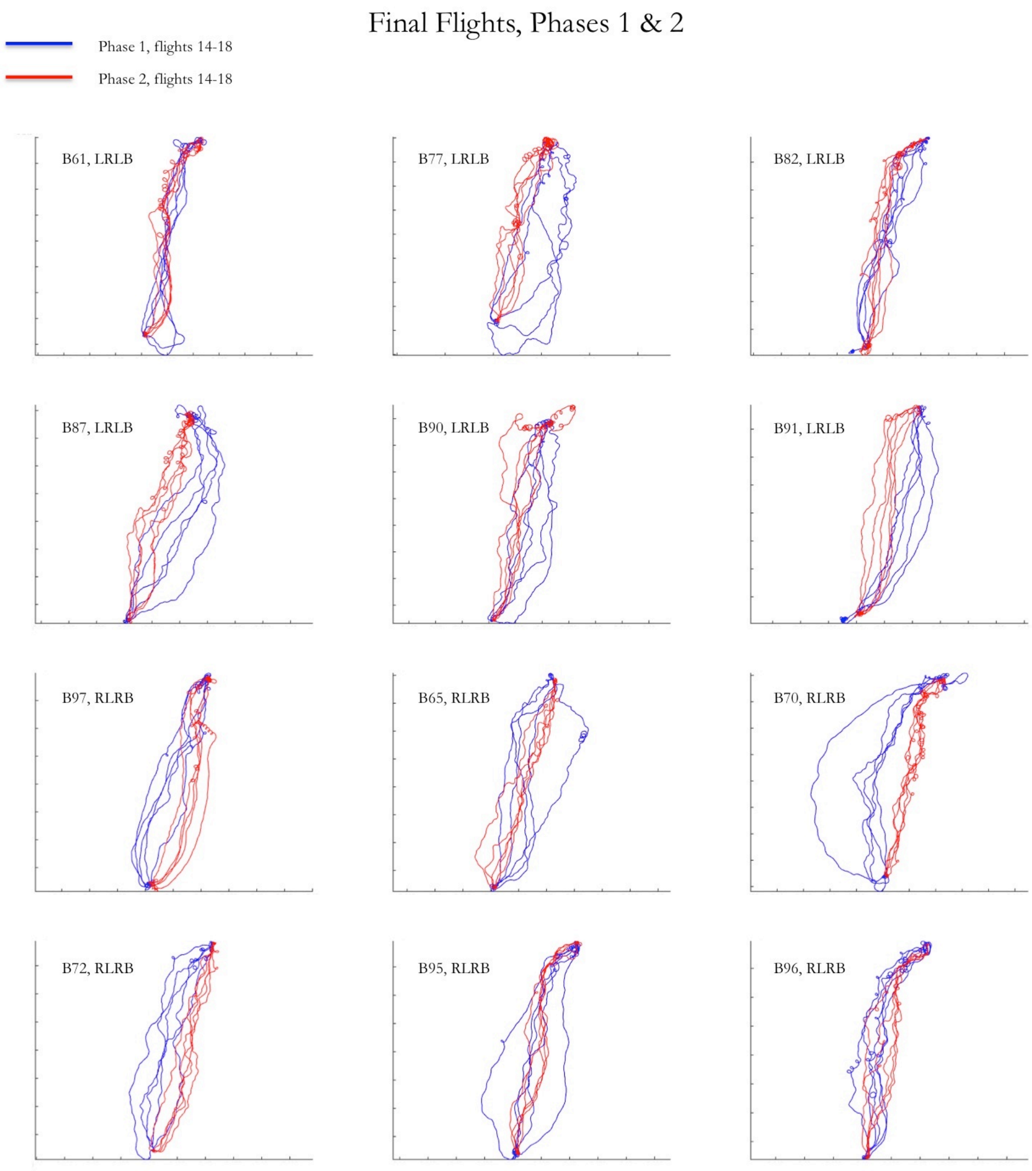

Figure S1. All birds' end-of-phase flights

This figure shows the traces of the last five flights in each of Phases 1 (blue) and 2 (red) for each of the 12 birds in the study. As can be seen, the flights of Phase 2 are generally flown over a different route from that flown during Phase 1. Neither group displayed a tendency to 'settle' back to their Phase 1 route in Phase 2.
